# The Herdecke questionnaire on quality of life (HLQ): Validation of factorial structure and development of a short form within a naturopathy treated in-patient collective

**DOI:** 10.1186/1477-7525-3-40

**Published:** 2005-07-08

**Authors:** Thomas Ostermann, Arndt Büssing, Andre-Michael Beer, Peter F Matthiessen

**Affiliations:** 1 Chair of Medical Theory and Complementary Medicine, Faculty of Medicine, University of Witten/Herdecke, Germany; 2 Department of Naturopathy, Blankenstein Hospital, Hattingen, Germany

**Keywords:** Questionnaires, Quality of life, short form, rheumatic diseases, naturopathy, Herdecke Questionnaire for Quality of Life;

## Abstract

**Background:**

Quality of life (QoL) of patients has become a central evaluation parameter that also acts as an aid for decisions related to treatment strategies particularly for patients with chronic illnesses. In Germany, one of the newer instruments attempting to measure distinct QoL aspects is the "Herdecke Questionnaire for Quality of Life" (HLQ). In this study, we aimed to validate the HLQ with respect to its factorial structure, and to develop a short form. The validation has been carried out in relation to other questionnaires including the SF-36 Health Survey, the Mood-Scale Bf-S, the Giessen Physical Complaints Questionnaire GBB-24 and McGill's Pain Perception Scale SES.

**Methods:**

Data for this study derived from a model project on the treatment of patients using naturopathy methods in Blankenstein Hospital, Hattingen. In total, 2,461 patients between the ages of 16 and 92 years (mean age: 58.0 ± 13.4 years) were included in this study. Most of the patients (62%) suffered from rheumatic diseases. Factorial validation of the HLQ, it's reliability and external consistency analysis and the development of a short form were carried out using the SPSS software.

**Results:**

Structural analysis of the HLQ-items pointed to a 6-factor model. The internal consistency of both the long and the short version is excellent (Cronbach's α is 0.935 for the HLQ-L and 0.862 for the HLQ-S). The highest reliability in the HLQ-L was obtained for the "Initiative Power and Interest" scale, the lowest for the 2-item scales "Digestive Well-Being" and the "Physical Complaints". However, the scales found by factor analysis herein were only in part congruent with the original 5-scale model which was approved a multitrait analysis approach. The new instrument shows good correlations with several scales of other relevant QoL instruments. The scales "Initiative Power and Interest", "Social Interaction", "Mental Balance", "Motility", "Physical Complaints", "Digestive Well-Being" sufficiently differentiate the diagnostic groups, particularly between the patients suffering on connective tissue and soft tissue disorders from those with metabolic and nutritional disorders or hypersensitivity reactions.

**Conclusion:**

Both the factorial validation and the development of a consistent short-form of the HLQ are important steps forward for researchers in the field of QoL who wish to use the HLQ as a reliable and valid instrument. The results indicate that the HLQ is a unique QoL-instrument that can be used for both in-patient and out-patient-treatment. However, to improve to profile of the HLQ, there is still the need for strengthening the Questionnaire in the dimensions of physical well-being. This is the subject of a separate ongoing study.

## Background

The consideration of "quality of life" (QoL) in clinical studies and various attempts to make this construct measurable to determine therapeutic success is an ongoing process. This is particularly the case in those therapeutic attempts that focus on integrative aspects of disease management that in turn offer holistic care including a variety of therapeutic directions. Here, the QoL has become a central evaluation parameter. It simultaneously acts as an aid for decisions on the choice of treatment strategy for chronically ill patients [1], which is obviously a challenging therapeutic aim, and is at least as significant as somatic parameters [2]. QoL has therefore become a leading criteria in many outcome studies alongside somatic and economic factors. In the course of this development, the concept of QoL is explicitly listed as outcome parameter in many medical societies' guidelines [3].

However, there are a variety of opinions regarding the factors that contribute to QoL. According to a WHO-definition, QoL relates to the physical, psychological and social well-being of an individual as laid out by formal health terms [4]. According to this definition, it is necessary to differentiate between a general and a health related QoL [5]. The former relates to aspects that exist independently from any particular disease (e.g. items such as "being spontaneous", or "feeling exhausted"), whereas the later focuses on particular characteristics of specific diseases (e.g. factors such as "walking distance" or "pain" in rheumatic diseases)

Despite the methodological difficulties involved in making QoL measurable, we have seen the development of numerous instruments for measuring disease specific aspects of QoL [6-8] in the recent past. An advantage of disease specific instruments is precise registration regarding strains and limitations of specific diseases rather than those of diseases in general. In addition, the course of clinical diseases can be more easily registered because of the development of disease-related questionnaires ("course of disease sensitivity" of questionnaires). The majority of current recommendations by health economists and clinical pharmacological associations include suggestions regarding the use of disease specific and general QoL questionnaires [9].

In Germany, one of the newer instruments attempting to measure general QoL with a distinct focus is the "Herdecke Questionnaire for Quality of Life" (HLQ is the German acronym of the phrase "Herdecke Questionnaire for Quality of Life") [10,11]. Clinical research projects have been reluctant to employ the HLQ although it was evaluated on a sample of healthy subjects, and that some reference values of clinical studies on different diseases do exist, and also despite of the fact that the HLQ has a very comprehensive understanding of the QoL problematic [12],. This is mainly because conclusive validation based on a large sample is still missing. To improve this situation, this study aimed to show the characteristics of the HLQ, to describe its external validation using other test instruments, and to develop a short form of the questionnaire.

## Methods

Data for this study derive from a model project on the treatment of patients using naturopathy methods in Blankenstein Hospital, Hattingen. To investigate the benefits and limits of naturopathic treatment in the field of in-patient care, the *Department of Naturopathy *was established as a model at the Blankenstein Hospital in Hattingen and was scientifically evaluated by the *Chair of Medical Theory and Complementary Medicine *of Witten/Herdecke University. This evaluation began on July 1^st ^1999 and was completed on March 31^th ^2003. It focused on the following question: "How does a three-week in-patient treatment with naturopathic methods affect the QoL of the patients, regarding a pre-post-comparison and a follow-up carried out after 6 months? Detailed information concerning this model project and its' scientific evaluation can be found in [13] and [14].

In total, 2,461 patients between 16 and 92 years (mean age 58.0 ± 13.4 years) were included in this study. The socio-demographic characteristics of the patients are shown in Table 1.

**Table 1:** Socio-demographic data of the patient population

		**male **(n = 507)		**female **(n = 1954)		**total **(n = 2461)	
**age**	mean	58,6		57,9		58,0	
	standard deviation	13,4		13,4		13,4	
	range	17–92		16–92		16–92	
		**n**	**%**	**n**	**%**	**n**	**%**

**age group**	under 18 years	1	0.2	3	0.2	4	0.2
	18–45 years	89	17.6	346	17.7	435	17.7
	45–60 years	162	32.0	676	34.6	838	34.1
	60–65 years	81	16.0	308	15.8	389	15.8
	65 and older	172	33.9	619	31.7	791	32.1
	no details available	2	0.4	2	0.1	4	0.2
**diagnostic groups**	connective tissue and soft tissue disorders	267	52.7	1305	66.8	1572	63.9
	chronic disorders of the respiratory system	35	6.9	60	3.1	95	3.9
	metabolic and nutritional disorders	92	18.1	133	6.8	225	9.1
	hypersensitivity reactions	8	1.6	46	2.4	54	2.2
	other indications	85	16.8	364	18.6	449	18.2
	no details available	20	3.9	46	2.4	66	2.7
**marital status**	single	56	11.0	178	9.1	234	9.5
	married	352	69.4	1055	54.0	1407	57.2
	living separated	7	1.4	34	1.7	41	1.7
	divorced	37	7.3	227	11.6	264	10.7
	widowed	36	7.1	399	20.4	435	17.7
	second marriage	12	2.4	28	1.4	40	1.6
	no details available	7	1.4	33	1.7	40	1.6
**education**	still at school	2	0.4	7	0.4	9	0.4
	no final exam	15	3.0	33	1.7	48	2.0
	special school exams	1	0.2	5	0.3	6	0.2
	secondary school exams other than GCSE	338	66.7	1191	61.0	1529	62.1
	GCSE ?	80	15.8	431	22.1	511	20,8
	A levels	60	11.8	201	10.3	261	10.6
	other	1	0.2	27	1.4	28	1.1
	no details available	10	2.0	59	3.0	69	2.8
**most recent profession**	worker	206	40.6	338	17.3	544	22.1
	employee/civil servant	212	41.7	941	48.2	1153	46.9
	self employed	42	8.3	94	4.8	136	5.5
	not working	15	3.0	242	12.4	257	10.4
	unclear	1	0.2	23	1.2	24	1.0
	no details available	31	6.1	316	16.2	347	14.1
**professional situation**	full-time professional	142	280	311	15.9	453	18.4
	part-time professional	19	3.8	288	14.8	307	12.4
	housewife/husband	12	2.4	484	24.8	496	20.2
	in training	4	0.8	13	0.7	17	0.7
	retired pre retired state	267	49.6	670	33.3	937	38.2
	unemployed	46	9.1	115	5.9	161	6.6
	no details available	17	3.4	73	3.7	90	3.7

Alongside the HLQ, other standardized questionnaires were used. These included the MOS-SF-36 Health Survey [15], Zerssen's Mood-Scale Bf-S [16], the Giessener Physical Complaints Questionnaire GBB-24 [17] and McGill's Pain Perception Scale SES [18].

The HLQ as referred to in this study uses 39 five-point likert scales ranging from 0 to 4 (agreement/disagreement or often/never). In contrast to the SF-36, the items are not defined by situations related to daily life and household situations (shopping, career situations, physical activity). As a result, the HLQ is very suitable for registering QoL particularly in monitoring the course of a disease or therapeutic intervention [19]. As an evaluation scheme, Schulte *et al*. [10] described 5 scales of the 39 item HLQ, unfortunately without any confirmation by factor analysis of the following areas: Physical Well-being (4 items), Vitality (9 items), Mental behavior (10 items), Presence of Personality (9 items), Social Environment (7 items). All scales are expressed in percentage values from 0 = lowest to 100 = highest QoL.

The main question of this study relates to the re-examination of the HLQ by means of a factor and reliability analysis and the explorative evaluation of the factors. External validation was performed by correlating the HLQ scales with those of the external test instruments: MOS-SF-36 Health Survey [15], Zerssen's Mood-Scale Bf-S [16], the Giessener Physical Complaints Questionnaire GBB-24 [17] and McGill's Pain Perception Scale SES [18].

Factor analysis was performed using principal components analysis with Varimax rotation on 35 of the 39 items. The items, #13 (avoided conflicts), #14 (behavior of others was unclear to me), #15 (was glad) and #29 (reduced sexual activity) were omitted following the positive preliminary results on the reliability of the HLQ by Kroez *et al*. [20]. To determine the internal consistency of the questionnaire, reliability analysis was performed using Cronbach's alpha. Both factor analysis and reliability analysis were performed for the long and the short version of the HLQ.

For the short form, only relevant items with a factorial weight of >0.6 were selected. This method of selection was originally suggested by Grimley [21] and has successfully been applied elsewhere [22,23].. Coefficients of determination (R-square) of short and long form scales were calculated to evaluate the proportion of variance of the original HLQ which can be explained by the short form.

Evaluation of responsiveness of the HLQ over a course of time was achieved by analyzing the change of HLQ-total score from the time of admission to the time of discharge by using a dependent t-test and calculation of Cohen's effect size (ES). Cohen's guidelines were used to classify the magnitude of effect sizes: 0.2 represents a small effect, 0.5 a moderate effect, and 0.8 a large effect.

The statistical data evaluation was performed using the SPSS Version 10.0 program packet.

## Results

The descriptive statistics of each item, the reliability parameters and the difficulty index are given in Table 2. Considering the high percentage of patients with chronic rheumatic diseases, an item-difficulty index between 0.26 (Item: "I suffered from physical pain") and 0.73 (Items "Family life was a burden" and "I felt over directed") can be regarded as sufficient. This also holds for item-total correlations with values between 0.27 and 0.69 (median: 0.55) for the original HLQ and between 0.32 and 0.61 (median: 0.46) for the short version (HLQ-S), These correlations are considered to be optimal ranges for psychological test instruments.

**Table 2:** Descriptive statistics and reliability parameters of HLQ-Items

**ItemNo.**	**Item**	**Mean**	**SD**	**Item-Diff. Index**	**loading**	**Cronbach's α **			**r**_**Item-total**_		**old Scale**
									
						**Total**		**Scale-wise**			
						long	short		long	short	
	**Initiative Power & Interest**							**0.885**			
10*	good ideas	2.26	0.94	0.57	0.747	0.933	0.852	0.875	0.54	0.49	**3**
07*	reacted spontaneously	2.13	1.08	0.53	0.640	0.934	0.855	0.880	0.47	0.42	**3**
11*	concerned	2.72	1.02	0.68	0.630	0.932	0.846	0.871	0.67	0.61	**3**
08*	decisive	2.44	0.91	0.61	0.617	0.934	0.852	0.876	0.54	0.48	**4**
12	put plans into action	2.09	0.91	0.52	0.572	0.933		0.877	0.58		**4**
25	difficult to take the initiative	2.33	1.12	0.58	0.535	0.932		0.873	0.65		**4**
36	enhanced personally	2.02	1.11	0.51	0.531	0.934		0.881	0.47		**4**
34	adapt to other people and situations	2.79	0.81	0.70	0.530	0.934		0.878	0.50		**3**
33	asserted in the environment	2.48	0.96	0.62	0.519	0.933		0.876	0.55		**4**
17	felt secure	2.36	0.95	0.59	0.481	0.932		0.874	0.66		**4**
21	future was clear	2.21	1.14	0.55	0.455	0.933		0.879	0.56		**4**
06	sought contact to others	2.27	1.10	0.57	0.453	0.935		0.884	0.41		**5**
30	felt enterprising/energetic	2.01	1.05	0.50	0.431	0.932		0.876	0.66		**3**
	**Social Interaction**							**0.812**			
16*	felt left out	2.79	1.07	0.70	0.697	0.933	0.850	0.775	0.57	0.52	**5**
27*	felt over-directed	2.90	1.10	0.73	0.636	0.933	0.853	0.780	0.57	0.51	**4**
20*	abandoned community life	2.51	1.12	0.63	0.631	0.932	0.847	0.766	0.66	0.59	**5**
18	family life was a burden	2.92	1.18	0.73	0.546	0.934		0.792	0.52		**5**
32	didn't feel comfortable in the company of others	2.31	1.10	0.58	0.532	0.934		0.808	0.46		**5**
28	convey feelings to other	2.63	0.92	0.66	0.496	0.933		0.788	0.57		**5**
05	anxious/fearful	2.42	1.20	0.61	0.461	0.933		0.797	0.56		**3**
	**Mental Balance**							**0.812**			
35*	nervous / irascibly	1.99	1.06	0.50	0.640	0.934	0.858	0.803	0.47	0.40	**3**
26*	well-balanced	2.01	0.97	0.50	0.600	0.932	0.850	0.770	0.67	0.61	**3**
19*	exhausted	1.24	0.94	0.31	0.567	0.933	0.849	0.782	0.59	0.56	**2**
09	could recover myself	1.57	0.99	0.39	0.557	0.933		0.784	0.55		**2**
31	tired	1.38	0.92	0.35	0.554	0.933		0.784	0.57		**2**
39	I happy	2.10	0.90	0.53	0.499	0.932		0.781	0.69		**3**
04	sleep was refreshing	1.55	1.05	0.39	0.356	0.935		0.809	0.42		**2**
	**Motility**							**0.781**			
22*	physically agile	1.95	1.02	0.49	0.789	0.935	0.854	0.708	0.40	0.43	**1**
24*	movement was light	1.84	1.07	0.46	0.786	0.934	0.851	0.673	0.47	0.46	**1**
38*	arms and legs felt heavy	1.36	1.07	0.34	0.668	0.935	0.855	0.571	0.39	0.42	**1**
37	powerful	1.47	0.95	0.37	0.509	0.933		0.482	0.56		**2**
	**Physical Complaints**							**0.692**			
02*	suffered from physical pain	1.04	0.97	0.26	0.726	0.936	0.859	*	0.27	0.32	**1**
01*	felt ill	1.09	0.91	0.27	0.705	0.935	0.853	*	0.42	0.44	**2**
	**Digestive Well-Being**							**0.621**			
03*	good appetite	2.71	1.12	0.68	0.763	0.935	0.857	*	0.37	0.38	**2**
23*	mealtimes were a burden	2.87	1.10	0.72	0.734	0.934	0.853	*	0.45	0.46	**2**

number of answered items ranged from 2,227 [min.] and 2,430 [max.]

* Short form Item

The results of the structural analysis of the HLQ-items yielded surprising results. The scales found by factor analysis (Table 2) were only partly congruent with the scalesin the original publication [10]. Instead, we found a new and stable 6-factor-model which fits better with the original data than the original 5-scale model derived by Schulte *et al*., which used a multitrait analysis approach (developed by Hays *et al*. [24]). This is underlined by a Kaiser-Meyer-Olkin measure of sampling adequacy of 0.957 and a highly significant Bartlett test of sphericity (p < 0.001). The cumulative variance explained by this model is 54.7%.

Correlation analysis (Table 3) of the earlier HLQ scale with the new scale revealed significant correlations between the scales "Social Environment (SOC)" and "Social Interaction (SOCI)" (r = 0.923 for the HLQ-L and r = 0.860 for the HLQ-S). Unfortunately, such clear correlation between an old HLQ scale with the unique factor of our current analysis was not found with the other scales. However, "physical well-being (PWB)" of the old HLQ correlated well with the new "motility (MOT)" scale (HLQ-L r = 0.929 resp. HLQ-S r = 0.958), while the old "vitality (VIT)" scale correlates with the "mental balance scale (MB)" (HLQ-L r = 0.840 resp. HLQ-S r = 0.681). The old scales "presence of personality (PERS)" and "mental balance (MEB)" are represented well by the new scale "initiative power and interest (IPI)" (See Table 3).

**Table 3:** Partial Correlation of HLQ-Scales with other instruments and with the HLQ-Scales (old adjusted for Gender and Age. Abbrev.: SF-36: PF-physical function, RP-role physical, BP-bodily pain, GH-general health, VT-vitality, SF-social function, RE-role emotional, MH-mental health, MCS-mental component summary, PCS-physical component summary; GBB: SE-severity of exhaustion, GS-gastric symptoms, LP-limb pain, and HS-heart symptoms;, Zerssen's Mood Scale Bf-S; SES: AFF-Affective Pain, SENS-SenSory Pain; HLQ-OLD: PWB-physical well-being, VIT-vitality, MEB-mental behaviour, PERS-presence of personality, SOC-Social Environment.

		**Initiative Power and Interest**		**Social Interaction**		**Mental Balance**		**Motility**		**Physical Complaints**		**Digestive Well-Being**	
		long	short	long	short	Long	short	long	short	long	Short	long	short
**SF-36**	**PF**	0.200	0.130	0.219	0.188	0.233	0.173	**0.551**	**0.580**	0.432	*	0.167	*
	**RP**	0.234	0.178	0.252	0.226	0.294	0.257	0.461	0.453	0.403	*	0.184	*
	**BP**	0.182	0.147	0.213	0.198	0.301	0.242	0.449	0.466	**0.688**	*	0.183	*
	**GH**	0.358	0.288	0.363	0.308	0.361	0.322	0.353	0.332	0.350	*	0.208	*
	**VT**	**0.562**	0.470	**0.532**	0.479	**0.668**	**0.582**	**0.541**	0.478	0.408	*	0.353	*
	**SF**	**0.543**	0.437	**0.632**	**0.589**	**0.550**	0.486	0.392	0.342	0.337	*	0.324	*
	**RE**	0.452	0.380	0.497	0.418	0.428	0.423	0.264	0.215	0.259	*	0.243	*
	**MH**	**0.650**	**0.543**	**0.686**	**0.605**	**0.719**	**0.690**	0.378	0.316	0.324	*	0.360	*
	**MCS**	**0.650**	**0.548**	**0.698**	**0.615**	**0.667**	**0.640**	0.294	0.213	0.245	*	0.348	*
	**PCS**	0.020	-0.012	0.022	0.025	0.087	0.024	0.489	0.530	0.497	*	0.089	*

**GBB 24**	**SE**	0.458	0.363	**0.513**	0.457	**0.626**	**0.546**	**0.559**	0.496	0.387	*	0.323	*
	**GS**	0.216	0.189	0.266	0.227 7	0.303	0.247	0.205	0.166	0.238	*	0.356	*
	**LP**	0.249	0.201	0.296	0.263	0.386	0.312	0.490	0.498	**0.514**	*	0.190	*
	**HS**	0.305	0.249	0.360	0.285	0.372	0.342	0.282	0.252	0.248	*	0.309	*

**Bf-S**		**0.655**	**0.564**	**0.652**	**0.599**	**0.630**	**0.581**	0.409	0.362	0.312	*	0.339	*

**SES**	**AFF**	0.289	0.231	0.314	0.277	0.371	0.325	0.374	0.372	0.506	*	0.183	*
	**SENS**	0.181	0.143	0.228	0.215	0.254	0.209	0.288	0.299	0.417	*	0.147	*

**HLQ-OLD**	**PWB**	0.357	0.270	0.335	0.333	0.489	0.412	**0.929**	**0.958**	**0.638**	*	0.259	*
	**VIT**	**0.577**	**0.502**	**0.590**	**0.523**	**0.840**	**0.681**	**0.611**	**0.517**	**0.560**	*	**0.661**	*
	**MEB**	**0.894**	**0.822**	**0.772**	**0.702**	**0.797**	**0.770**	0.471	0.401	0.278	*	0.412	*
	**PERS**	**0.926**	**0.779**	**0.760**	**0.734**	**0.628**	**0.584**	0.404	0.354	0.226	*	0.342	*
	**SOC**	**0.748**	**0.697**	**0.923**	**0.860**	**0.600**	**0.573**	0.358	0.346	0.248	*	0.346	*

* values of the long version are identical with short version

The internal consistency of the instruments (HLQ-L and HLQ-S), both for the total score (Cronbach's α is 0.935 for the HLQ-L and 0.862 for the HLQ-S) as well as for the subscales of the HLQ-L (Cronbach's α between 0.621 and 0.885) can be regarded as being excellent. The highest alpha reliability in the HLQ-L was obtained for the "Initiative Power and Interest" scale, the lowest for the 2-item scales "Digestive Well-Being" (0.621) and "Physical Complaints" (0.692).

The mean difference between the scales of the HLQ-S and the HLQ-L for all patients is between 1.20 ("Initiative Power and Interest ") and 2.24 points ("Social interaction") on a percentage scale. The absolute differences are clustered in groups and are given in Table 4. Although there is a low overall mean difference, absolute differences greater than 10 percent range between 17.9% ("Initiative Power and Interest") and 26.8% ("Social interaction"). However, with correlation coefficients ranging from 0.899 to 0.964, the proportion of variance of the HLQ-L can be explained by the short form ranges between 79% and 93% and thus can be regarded as an adequate proportion for a short version.

**Table 4:** Comparison of the HLQ-L and the HLQ-S.

	**Difference of means**	**Percentage of Patients with a mean difference D**					**Correlation**	**Explained Variance**
				
		< 3	3< D <7	7< D <10	10<D< 20	>20		
**Initiative Power and Interest**	1.20	32.9%	33.6%	15.6%	15.8%	2.1%	0.899	81%
**Social Interaction**	2.24	25.9%	27.3%	20.1%	22.2%	4.6%	0.909	83%
**Mental Balance**	1.43	27.8%	28.3%	19.6%	20.4%	3.9%	0.888	79%
**Motility**	1.43	46.5%	29.7%	11.9%	11.1%	0.8%	0.964	93%
**Physical Complaints**	*	*	*	*	*	*	*	*
**Digestive well-Being**	*	*	*	*	*	*	*	*

* values of the long version are identical with short version

The correlation of the HLQ with other test instruments is shown in Table 5. There are acceptable correlations with r> 0.5 between the mental-health associated scales from the HLQ with those of the other instruments, for example, the "mental health"-Scale of the SF-36. In detail, the scales "Initiative Power and Interest", "Social Interaction" and "Mental Balance" of the HLQ correlate well with "mental health" and the "mental component summary", "Social Functionand "Vitality" of the SF-36 and Zerssens Bf-S Mood-Scale. The "motility" scale of the HLQ correlates with "physical function" and "vitality" of the SF-36, with the "severity of exhaustion" of the Giessener Physical Complaints Questionnaire GBB 24, and somewhat weaker with the "role physical", "bodily pain" and "physical component summary" scales of SF-36 and "limp pain" of the GBB 24. The "physical complaints" subscale of the HLQ correlates well with "bodily pain" of the SF-36 and its "physical component summary" scale, and also with the "affection pain" subscale of McGill's Pain Perception Scales SES. Among the SF-36 scales, the factor "general health" is not represented by the HLQ scales. The factors, "gastric symptoms" and the "heart symptoms" from the GBB 24 scales and "sensory pain" from the SES are not represented by the HLQ.

**Table 5:** HLQ-scales (Mean ± SD)) of patients separated into diagnostic-, age-and gender specific groups.

	**age**	**gender**	**n**	**Initiative Power and Interest**	**Social Interaction**	**Mental Balance**	**Motility**	**Physical Complaints**	**Digestive Well-Being**
**Connective tissue and soft tissue disorders**	18–45	M	43	56.4 ± 15.2	67.4 ± 18.9	42.8 ± 17,2	42.0 ± 18.4	25.3 ± 15.8	73.3 ± 20.3
		F	167	54.0 ± 15.2	64.2 ± 19.4	36.8 ± 15.8	38.7 ± 19.0	25.6 ± 17.4	67.4 ± 22.4
	45–60	M	100	60.7 ± 16.9	69.9 ± 18.8	46.4 ± 16.0	42.8 ± 17.9	25.9 ± 14.0	77.0 ± 19.5
		F	483	54.5 ± 15.1	60.2 ± 18.9	37.9 ± 15.1	37.0 ± 18.4	21.6 ± 16.9	67.6 ± 23.3
	60–65	M	36	64.9 ± 16.0	73.1 ± 17.9	47.4 ± 19.4	41.3 ± 19.6	29.9 ± 17.7	79.9 ± 21.6
		F	208	61.0 ± 15.5	66.9 ± 18.0	41.9 ± 15.0	40.3 ± 17.9	23.7 ± 16.3	74.3 ± 19.8
	> 65	M	86	60.7 ± 16.9	70.7 ± 18.6	48.0 ± 16.5	36.9 ± 20.9	20.1 ± 19.2	75.1 ± 23.8
		F	437	61.0 ± 16.2	69.2 ± 18.0	46.1 ± 16.8	38.3 ± 21.4	19.4 ± 17.6	70.0 ± 24.7

**Chronic disorders of the respiratory system**	18–45	M	5	60.4 ± 19.2	70.7 ± 14.6	46.4 ± 20.0	42.5 ± 24.6	27.5 ± 31.1	85.0 ± 22.4
		F	12	50.2 ± 9.0	62.2 ± 23.2	34.8 ± 12.1	49.0 ± 17.6	35.4 ± 19.1	59.4 ± 30.7
	45–60	M	2	58.7 ± 14.9	73.2 ± 12.6	58.9 ± 7.6	43.8 ± 8.9	37.5 ± 0.00	81.3 ± 26.5
		F	14	61.9 ± 11.0	66.6 ± 14.4	37.9 ± 10.3	39.4 ± 18.7	27.9 ± 17.0	65.2 ± 18.5
	60–65	M	6	76.6 ± 6.9	72.0 ± 6.5	47.6 ± 10.5	48.9 ± 28.1	25.0 ± 17.7	85.4 ± 12.3
		F	12	55.1 ± 17.6	67.7 ± 16.4	37.9 ± 16.1	41.7 ± 16.7	31.8 ± 20.4	59.4 ± 35.0
	> 65	M	22	57.0 ± 18.3	67.7 ± 20.1	45.9 ± 18.2	43.2 ± 23.1	29.4 ± 22.7	65.3 ± 27.0
		F	22	56.0 ± 15.3	67.2 ± 18.8	42.9 ± 14.2	48.6 ± 22.4	31.8 ± 24.9	69.3 ± 21.4

**Metabolic and nutritional disorders**	18–45	M	15	54.4 ± 16.2	64.8 ± 20.9	48.1 ± 18.5	53.9 ± 18.4	56.7 ± 29.8	77.5 ± 19.0
		F	24	58.1 ± 14.9	69.3 ± 21.8	40.7 ± 13.6	45.3 ± 20.5	42.4 ± 28.4	64.1 ± 25.4
	45–60	M	25	55.9 ± 16.3	71.4 ± 18.3	45.8 ± 14.8	40.8 ± 16.4	43.0 ± 27.3	75.5 ± 21.5
		F	40	59.4 ± 16.7	65.9 ± 18.4	44.1 ± 17.6	46.3 ± 20.4	39.1 ± 24.1	72.8 ± 24.0
	60–65	M	20	70.9 ± 12.6	81.6 ± 13.0	56.7 ± 14.8	53.7 ± 18.4	38.1 ± 21.3	80.0 ± 15.9
		F	16	67.9 ± 10.4	74.3 ± 15.2	54.2 ± 10.7	44.9 ± 21.8	39.1 ± 28.5	83.6 ± 15.6
	> 65	M	32	66.2 ± 15.0	75.8 ± 14.8	53.0 ± 14.6	49.5 ± 22.3	41.8 ± 19.7	79.3 ± 19.5
		F	53	62.1 ± 17.3	70.6 ± 16.6	50.9 ± 19.3	45.3 ± 23.2	30.9 ± 25.2	76.2 ± 20.3

**Hypersensitivity and allergic reactions**	18–45	M	4	59.1 ± 10.5	68.8 ± 14.7	53.6 ± 28.1	64.1 ± 16.4	28.1 ± 12.0	68.7 ± 26.0
		F	16	63.3 ± 17.1	73.8 ± 17.8	38.3 ± 16.2	46.9 ± 20.9	45.3 ± 29.5	67.2 ± 26.6
	45–60	M	2	55.8 ± 35.4	62.5 ± 42.9	51.8 ± 37.9	43.8 ± 44.2	25.0 ± 35.4	87.5 ± 17.7
		F	15	58.1 ± 14.6	69.9 ± 16.2	42.3 ± 16.1	48.8 ± 17.9	37.5 ± 22.2	61.7 ± 25.6
	60–65	M	-	-	-	-	-	-	-
		F	8	51.8 ± 18.1	62.1 ± 22.4	32.5 ± 11.1	52.3 ± 17.0	28.1 ± 16.0	62.5 ± 25.0
	> 65	M	-	-	-	-	-	-	-
		F	6	71.3 ± 12.9	77.0 ± 7.1	55.2 ± 22.3	60.4 ± 21.2	22.9 ± 12.3	83.3 ± 18.8

**other indications**	18–45	M	18	47.6 ± 17.5	62.9 ± 15.6	40.7 ± 16.1	48.6 ± 18.4	35.4 ± 16.7	63.2 ± 23.3
		F	122	53.8 ± 17.0	60.1 ± 19.8	35.2 ± 16.3	46.6 ± 21.5	35.9 ± 24.0	59.8 ± 24.2
	45–60	M	28	57.2 ± 16.8	64.3 ± 25.0	41.8 ± 16.1	40.6 ± 19.1	34.8 ± 19.1	67.4 ± 27.1
		F	111	53.3 ± 17.0	58.1 ± 18.4	35.1 ± 14.1	43.9 ± 19.5	31.3 ± 19.6	59.8 ± 25.1
	60–65	M	15	55.3 ± 16.8	69.5 ± 14.1	44.8 ± 16.8	45.4 ± 18.8	40.8 ± 28.9	71.7 ± 21.9
		F	50	53.4 ± 18.1	58.4 ± 17.3	40.4 ± 14.7	49.6 ± 19.0	31.5 ± 20.7	63.0 ± 26.5
	> 65	M	23	65.8 ± 18.8	72.5 ± 16.7	52.8 ± 16.5	52.5 ± 24.6	34.2 ± 27.5	83.2 ± 19.4
		F	78	59.0 ± 17.6	69.0 ± 18.9	47.8 ± 16.9	50.6 ± 21.8	30.2 ± 27.0	70.7 ± 26.6

According to the diagnostic spectrum (Table 5), the values of the scale "Motility (MOT)" and "Physical Complaints (PHY)" show particularly low values in patients suffering from rheumatic diseases. Also, in contrast with other scales of the HLQ, these two appear to have little correlation with age, which indicates a suitable discriminatory power of the HLQ considering age and different types of disease.

The results from the responsiveness analysis are presented in Table 6. We found a high sensitivity of the HLQ-scales to change within the treatment with particularly high significant changes in the mean and calculated effect sizes between 0.39 (Digestive Well Being) and 0.92 (Mental Balance).

**Table 6:** Responsiveness of HLQ-scales measured with Cohen's effect size.

	**Mean Difference [95% CI] (Admission-Discharge)**	**t-value**	**N**	**Effect-Size *ES***
**Initiative Power and Interest**	8.1 [7.4; 8.7]	24.89	2064	0.55
**Social Interaction**	11.0 [10.3; 11.7]	30.18	2062	0.67
**Mental Balance**	15.8 [15.1; 16.5]	41.75	2066	0.92
**Motility**	11.4 [10.6; 12.3]	25.49	2050	0.57
**Physical Complaints**	21.7 [20.6; 22.8]	39.96	2022	0.89
**Digestive well-Being**	9.8 [8.7; 10.9]	17.78	2053	0.39

## Discussion

The aim of our study was to confirm the structure and consistency of the HLQ. Surprisingly, we found that the original scales presented earlier [10] were not in accordance with the results of this factor analysis. However, the scales "IPI-Initiative Power and Interest", "SOCI – Social Interaction", "MB – Mental Balance", "MOT – Motility", "PHY-Physical Complaints", "DWB – Digestive Well-Being" show a good reliability and sufficiently differentiate the diagnostic groups, especially between those patients suffering with connective tissue and soft tissue disorders from those with metabolic and nutritional disorders or hypersensitivity reactions.

Although the HLQ sub-scales "Initiative Power and Interest", "Social Interaction" and "Mental Balance" of the HLQ correlate well with the corresponding SF-36 scales and with Zerssens Bf-S Mood-Scale, and thus indicate that these qualities share several interconnections, our findings also showed that the HLQ provides several aspects of health such as "Appetite and Digestive Affections" which are not well covered by existing QoL-measures. Nevertheless, with only two items, the subscale "digestive well-being" has to be strengthened by additional items. This is also true for the scale related to physical complaints and pain. With correlation values of 0.11 (physical total scale of the SF-36) and 0.29 (sensory pain SES), it is quite obvious that this scale is deficient and needs an upgrade in respect to quality and number of items.

As, according to [25] internal consistency reliability is a poor predictor of responsiveness, we measured the responsiveness of the HLQ directly using Cohen's effect size. Together with the highly significant results of the t-test statistics and being aware of the methodological limitations which are immanent in obtaining results on a questionnaires responsiveness by means of effect sizes [26], we can nevertheless conclude that the HLQ shows sufficient responsiveness for the use in a clinical setting.

In our opinion, the HLQ is more sensitive to health changes brought about by Complementary Therapies including anthroposophic medicine or homeopathy. This does not mean that the HLQ is only suitable for such therapies. Although, there is a trend to consider QoL-questionnaires being specific for special complementary therapies such as mistletoe treatment in cancer patients [27], we do not favor such labels, as this might result in an inflation of "new" QoL-measures for each new therapeutic situation [28].

QoL is a multidimensional construct composed of functional, physical, emotional, social and spiritual well-being [29,30] with, several interconnections between distinct constructs of well-being. The HLQ scales "Social Interaction", "Mental Balance", "Motility", and "Physical Complaints" share similarities with the these constructs, but highlights two further significant topics, i.e. "Initiative Power and Interest" and "Digestive Well-Being". The highly relevant topic of spirituality and illness is addressed in another instrument developed by our group, the SpREUK questionnaire, with its sub-scales "Search for meaningful support", "Positive interpretation of disease", "Trust in external guidance", "Support through spirituality/religiosity" [22,31,32].

Our evaluation indicates an adequate representation of aspects like "mental well-being" and "depression" which are essential in defining QoL, and shows special features of the HLQ that highlights its' uniqueness in the group of generic QoL-measures. Particularly in clinical studies in which, because of feasibility or patient compliance the use of huge psychometric test batteries is inappropriate, the HLQ now serves as a economic test-instrument. To conclude, we can state that this study presents necessary foundations and developments for existing and future studies that wish to use the HLQ as a reliable and valid instrument.

## Authors' contributions

TO performed the statistical analysis, was responsible for the methodological setting of the study and has written most parts of the manuscript, AB has written some parts of manuscript, AMB is director of the study centre and was the clinical supervisor, PFM conceived and designed the study. All authors have read and approved the final manuscript.
